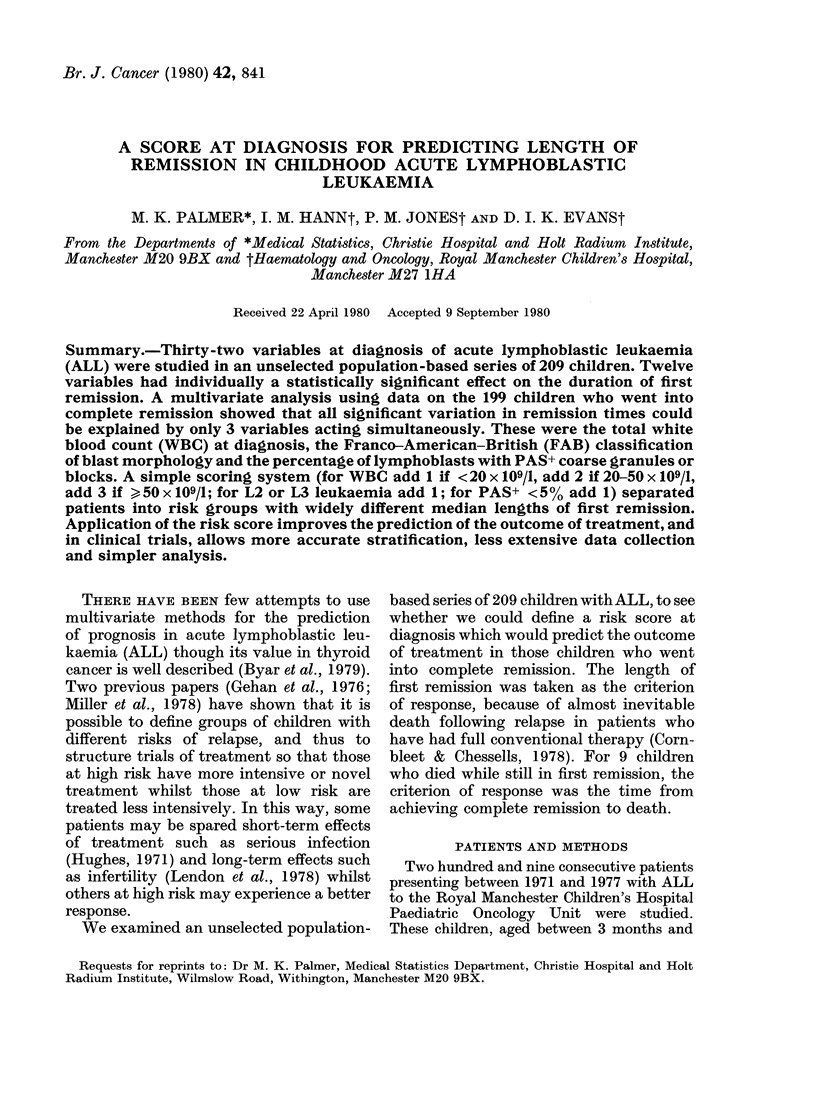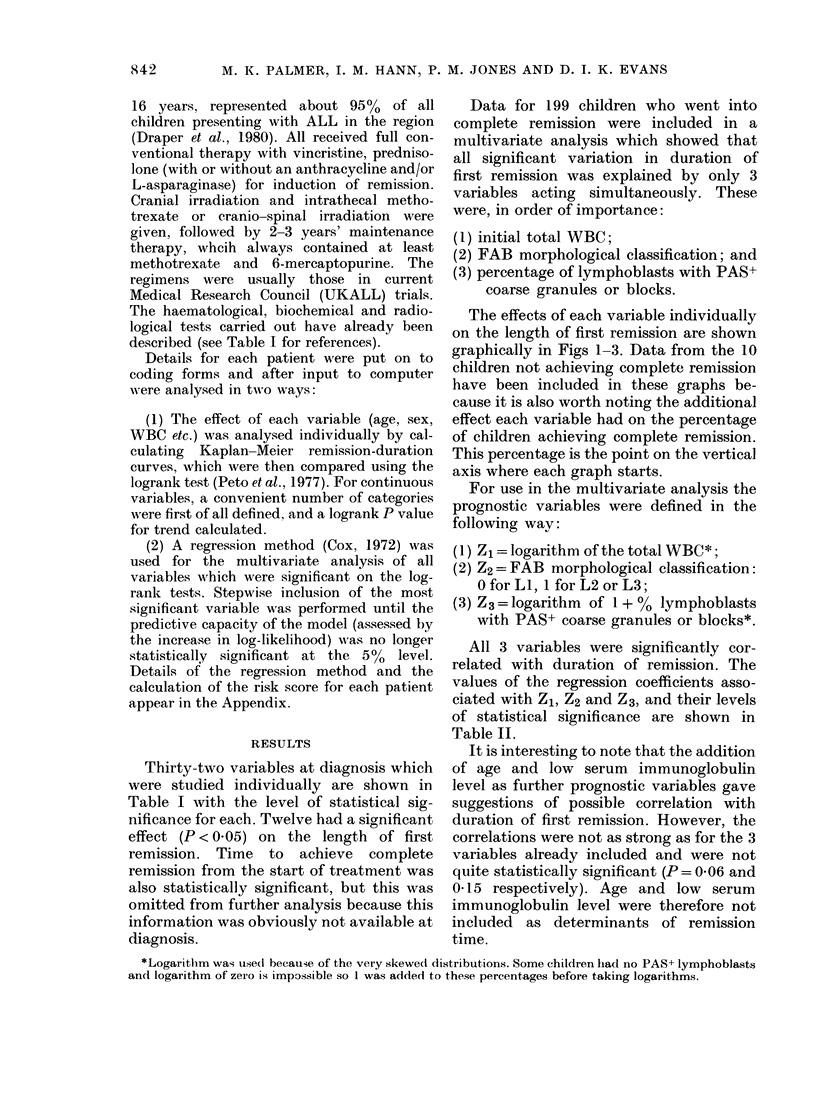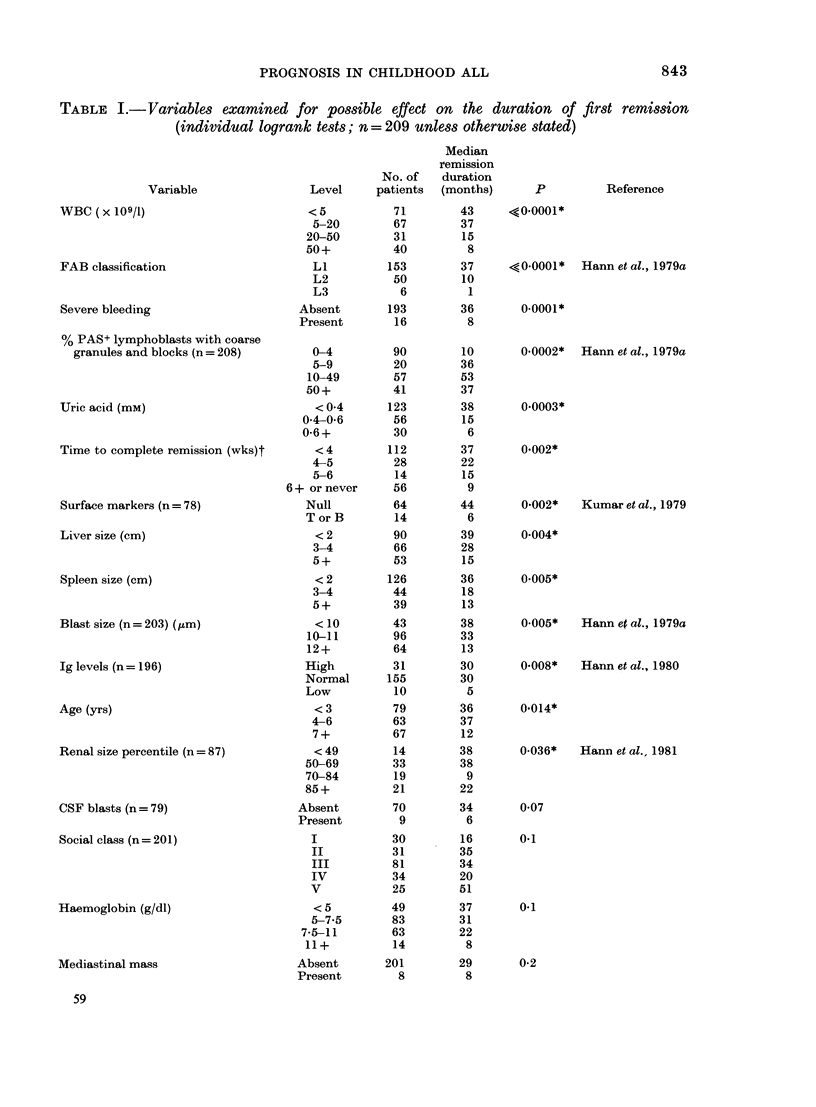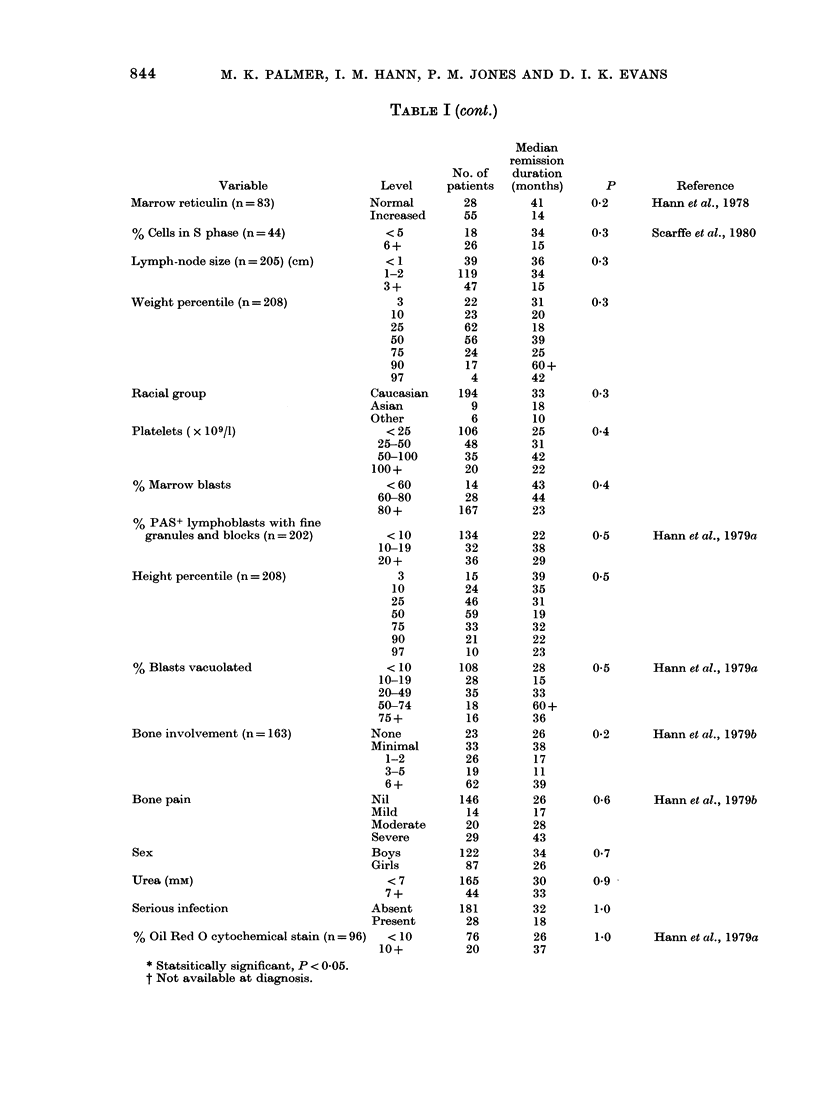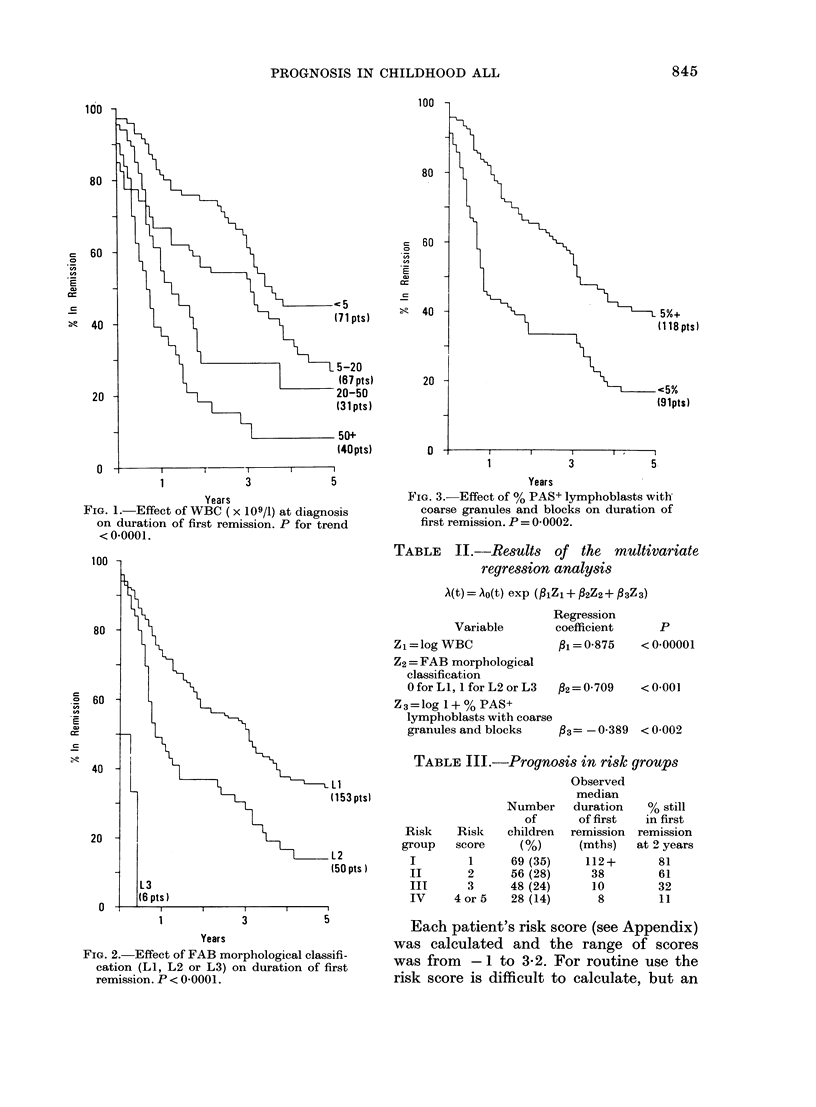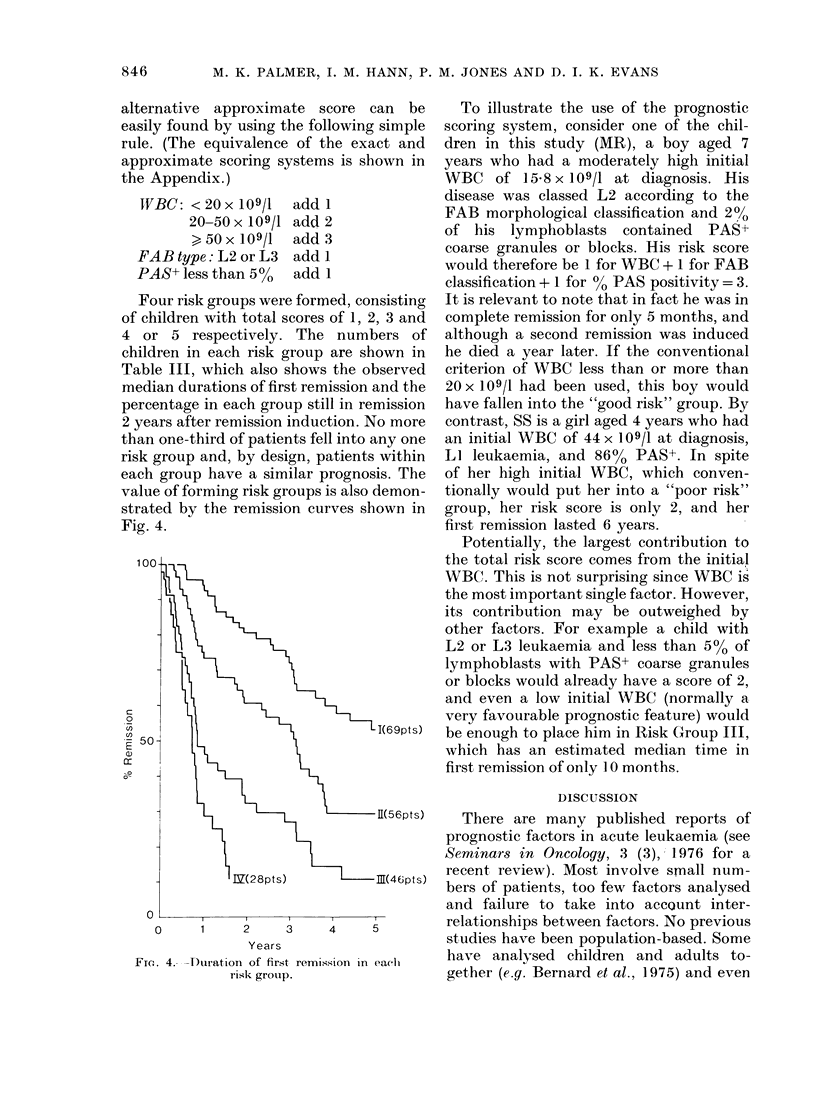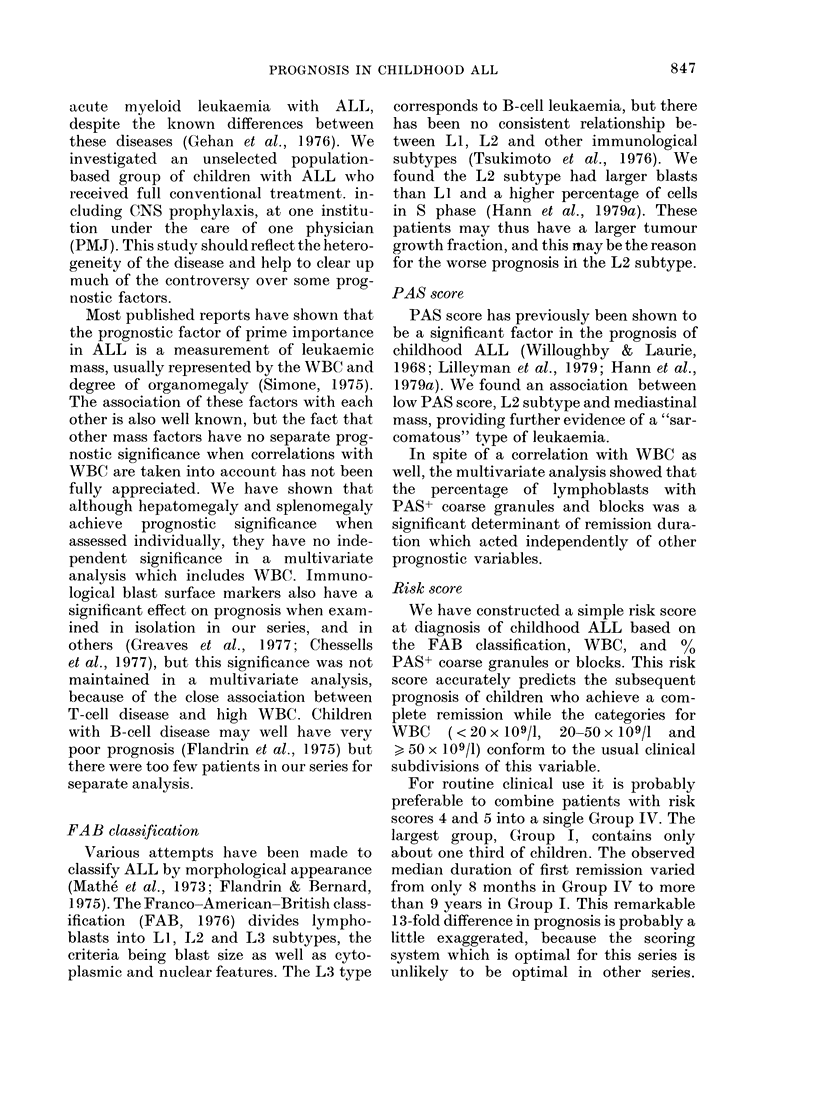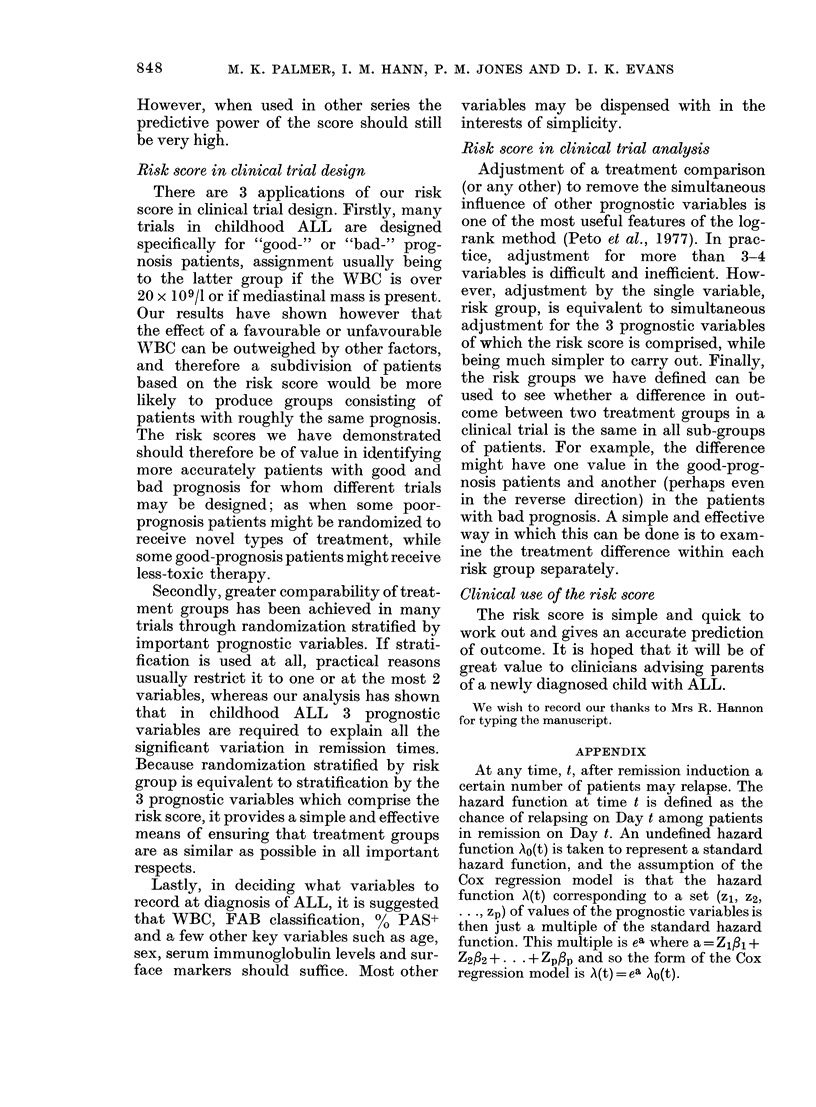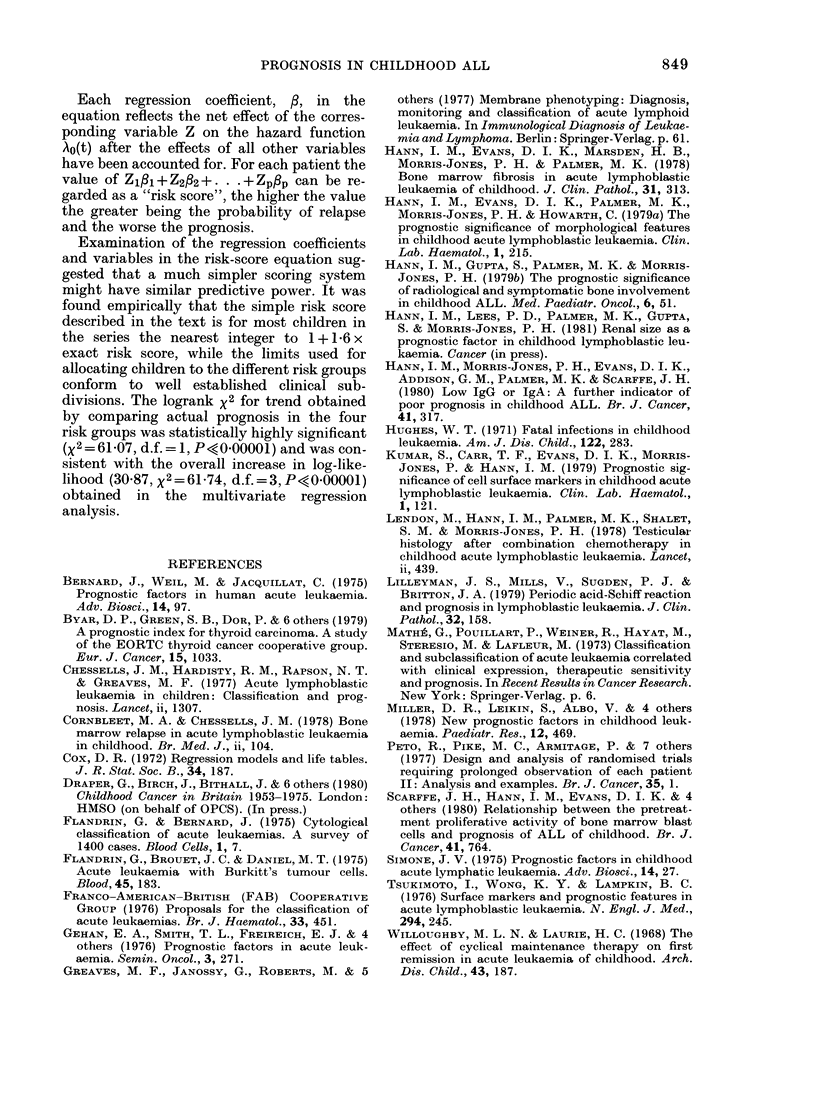# A score at diagnosis for predicting length of remission in childhood acute lymphoblastic leukaemia.

**DOI:** 10.1038/bjc.1980.331

**Published:** 1980-12

**Authors:** M. K. Palmer, I. M. Hann, P. M. Jones, D. I. Evans

## Abstract

Thirty-two variables at diagnosis of acute lymphoblastic leukaemia (ALL) were studied in an unselected population-bases series of 209 children. Twelve variables had individually a statistically significant effect on the duration of first remission. A multivariate analysis using data on the 199 children who went into complete remission showed that all significant variation in remission times could be explained by only 3 variables acting simultaneously. These were the total white blood count (WBC) at diagnosis, the Franco-American-British (FAB) classification of blast morphology and the percentage of lymphoblasts with PAS+ coarse granules or blocks. A simple scoring system (for WBC add 1 if less than 20 X 10(9)/1, add 2 if 20 - 50 X 10(9)/1, add 3 if greater than or equal to 50 X 10(9)/1; for L2 or L3 leukaemia add 1; for PAS+ less than 5% add 1) separated patients into risk groups with widely different median lengths of first remission. Application of the risk score improves the prediction of the outcome of treatment, and the clinical trials, allows more accurate stratification, less extensive data collection and simpler analysis.


*Br. J. Cancer* (1980) **42,** 841

### A SCORE AT DIAGNOSIS FOR PREDICTING LENGTH OF REMISSION IN CHILDHOOD ACUTE LYMPHOBLASTIC LEUKAEMIA

M. K. PALMER*, I. M. HANN†, P. M. JONES† AND D. I. K. EVANS†

*From the Departments of *Medical Statistics, Christie Hospital and Holt Radium Institute, Manchester M20 9BX and †Haematology and Oncology, Royal Manchester Children's Hospital, Manchester M27 1HA*

Received 22 April 1980 Accepted 9 September 1980

**Summary.—Thirty-two variables at diagnosis of acute lymphoblastic leukaemia (ALL) were studied in an unselected population-based series of 209 children. Twelve variables had individually a statistically significant effect on the duration of first remission. A multivariate analysis using data on the 199 children who went into complete remission showed that all significant variation in remission times could be explained by only 3 variables acting simultaneously. These were the total white blood count (WBC) at diagnosis, the Franco-American-British (FAB) classification of blast morphology and the percentage of lymphoblasts with PAS$^+$ coarse granules or blocks. A simple scoring system (for WBC add 1 if $<20\times10^9$/l, add 2 if 20–50 $\times10^9$/l, add 3 if $\geqslant50\times10^9$/l; for L2 or L3 leukaemia add 1; for PAS$^+$ $<5\%$ add 1) separated patients into risk groups with widely different median lengths of first remission. Application of the risk score improves the prediction of the outcome of treatment, and in clinical trials, allows more accurate stratification, less extensive data collection and simpler analysis.**

THERE HAVE BEEN few attempts to use multivariate methods for the prediction of prognosis in acute lymphoblastic leukaemia (ALL) though its value in thyroid cancer is well described (Byar *et al.*, 1979). Two previous papers (Gehan *et al.*, 1976; Miller *et al.*, 1978) have shown that it is possible to define groups of children with different risks of relapse, and thus to structure trials of treatment so that those at high risk have more intensive or novel treatment whilst those at low risk are treated less intensively. In this way, some patients may be spared short-term effects of treatment such as serious infection (Hughes, 1971) and long-term effects such as infertility (Lendon *et al.*, 1978) whilst others at high risk may experience a better response.

We examined an unselected population-based series of 209 children with ALL, to see whether we could define a risk score at diagnosis which would predict the outcome of treatment in those children who went into complete remission. The length of first remission was taken as the criterion of response, because of almost inevitable death following relapse in patients who have had full conventional therapy (Cornbleet & Chessells, 1978). For 9 children who died while still in first remission, the criterion of response was the time from achieving complete remission to death.

### PATIENTS AND METHODS

Two hundred and nine consecutive patients presenting between 1971 and 1977 with ALL to the Royal Manchester Children's Hospital Paediatric Oncology Unit were studied. These children, aged between 3 months and

Requests for reprints to: Dr M. K. Palmer, Medical Statistics Department, Christie Hospital and Holt Radium Institute, Wilmslow Road, Withington, Manchester M20 9BX.

16 years, represented about 95% of all children presenting with ALL in the region (Draper *et al.*, 1980). All received full conventional therapy with vincristine, prednisolone (with or without an anthracycline and/or L-asparaginase) for induction of remission. Cranial irradiation and intrathecal methotrexate or cranio-spinal irradiation were given, followed by 2–3 years' maintenance therapy, whcih always contained at least methotrexate and 6-mercaptopurine. The regimens were usually those in current Medical Research Council (UKALL) trials. The haematological, biochemical and radiological tests carried out have already been described (see Table I for references).

Details for each patient were put on to coding forms and after input to computer were analysed in two ways:

(1) The effect of each variable (age, sex, WBC *etc.*) was analysed individually by calculating Kaplan–Meier remission-duration curves, which were then compared using the logrank test (Peto *et al.*, 1977). For continuous variables, a convenient number of categories were first of all defined, and a logrank $P$ value for trend calculated.

(2) A regression method (Cox, 1972) was used for the multivariate analysis of all variables which were significant on the logrank tests. Stepwise inclusion of the most significant variable was performed until the predictive capacity of the model (assessed by the increase in log-likelihood) was no longer statistically significant at the 5% level. Details of the regression method and the calculation of the risk score for each patient appear in the Appendix.

### RESULTS

Thirty-two variables at diagnosis which were studied individually are shown in Table I with the level of statistical significance for each. Twelve had a significant effect ($P < 0{\cdot}05$) on the length of first remission. Time to achieve complete remission from the start of treatment was also statistically significant, but this was omitted from further analysis because this information was obviously not available at diagnosis.

Data for 199 children who went into complete remission were included in a multivariate analysis which showed that all significant variation in duration of first remission was explained by only 3 variables acting simultaneously. These were, in order of importance:

(1) initial total WBC;
(2) FAB morphological classification; and
(3) percentage of lymphoblasts with $PAS^+$ coarse granules or blocks.

The effects of each variable individually on the length of first remission are shown graphically in Figs 1–3. Data from the 10 children not achieving complete remission have been included in these graphs because it is also worth noting the additional effect each variable had on the percentage of children achieving complete remission. This percentage is the point on the vertical axis where each graph starts.

For use in the multivariate analysis the prognostic variables were defined in the following way:

(1) $Z_1$ = logarithm of the total WBC*;
(2) $Z_2$ = FAB morphological classification: 0 for L1, 1 for L2 or L3;
(3) $Z_3$ = logarithm of $1 + \%$ lymphoblasts with $PAS^+$ coarse granules or blocks*.

All 3 variables were significantly correlated with duration of remission. The values of the regression coefficients associated with $Z_1$, $Z_2$ and $Z_3$, and their levels of statistical significance are shown in Table II.

It is interesting to note that the addition of age and low serum immunoglobulin level as further prognostic variables gave suggestions of possible correlation with duration of first remission. However, the correlations were not as strong as for the 3 variables already included and were not quite statistically significant ($P = 0{\cdot}06$ and $0{\cdot}15$ respectively). Age and low serum immunoglobulin level were therefore not included as determinants of remission time.

*Logarithm was used because of the very skewed distributions. Some children had no $PAS^+$ lymphoblasts and logarithm of zero is impossible so 1 was added to these percentages before taking logarithms.

TABLE I.—*Variables examined for possible effect on the duration of first remission (individual logrank tests; n* = 209 *unless otherwise stated)*

| Variable | Level | No. of patients | Median remission duration (months) | *P* | Reference |
|---|---|---|---|---|---|
| WBC (× $10^9$/l) | < 5 | 71 | 43 | ⩽0·0001* | |
| | 5–20 | 67 | 37 | | |
| | 20–50 | 31 | 15 | | |
| | 50+ | 40 | 8 | | |
| FAB classification | L1 | 153 | 37 | ⩽0·0001* | Hann *et al.*, 1979*a* |
| | L2 | 50 | 10 | | |
| | L3 | 6 | 1 | | |
| Severe bleeding | Absent | 193 | 36 | 0·0001* | |
| | Present | 16 | 8 | | |
| % $PAS^+$ lymphoblasts with coarse granules and blocks (n = 208) | 0–4 | 90 | 10 | 0·0002* | Hann *et al.*, 1979*a* |
| | 5–9 | 20 | 36 | | |
| | 10–49 | 57 | 53 | | |
| | 50+ | 41 | 37 | | |
| Uric acid (mM) | < 0·4 | 123 | 38 | 0·0003* | |
| | 0·4–0·6 | 56 | 15 | | |
| | 0·6+ | 30 | 6 | | |
| Time to complete remission (wks)† | < 4 | 112 | 37 | 0·002* | |
| | 4–5 | 28 | 22 | | |
| | 5–6 | 14 | 15 | | |
| | 6+ or never | 56 | 9 | | |
| Surface markers (n = 78) | Null | 64 | 44 | 0·002* | Kumar *et al.*, 1979 |
| | T or B | 14 | 6 | | |
| Liver size (cm) | < 2 | 90 | 39 | 0·004* | |
| | 3–4 | 66 | 28 | | |
| | 5+ | 53 | 15 | | |
| Spleen size (cm) | < 2 | 126 | 36 | 0·005* | |
| | 3–4 | 44 | 18 | | |
| | 5+ | 39 | 13 | | |
| Blast size (n = 203) (μm) | < 10 | 43 | 38 | 0·005* | Hann *et al.*, 1979*a* |
| | 10–11 | 96 | 33 | | |
| | 12+ | 64 | 13 | | |
| Ig levels (n = 196) | High | 31 | 30 | 0·008* | Hann *et al.*, 1980 |
| | Normal | 155 | 30 | | |
| | Low | 10 | 5 | | |
| Age (yrs) | < 3 | 79 | 36 | 0·014* | |
| | 4–6 | 63 | 37 | | |
| | 7+ | 67 | 12 | | |
| Renal size percentile (n = 87) | < 49 | 14 | 38 | 0·036* | Hann *et al.*, 1981 |
| | 50–69 | 33 | 38 | | |
| | 70–84 | 19 | 9 | | |
| | 85+ | 21 | 22 | | |
| CSF blasts (n = 79) | Absent | 70 | 34 | 0·07 | |
| | Present | 9 | 6 | | |
| Social class (n = 201) | I | 30 | 16 | 0·1 | |
| | II | 31 | 35 | | |
| | III | 81 | 34 | | |
| | IV | 34 | 20 | | |
| | V | 25 | 51 | | |
| Haemoglobin (g/dl) | < 5 | 49 | 37 | 0·1 | |
| | 5–7·5 | 83 | 31 | | |
| | 7·5–11 | 63 | 22 | | |
| | 11+ | 14 | 8 | | |
| Mediastinal mass | Absent | 201 | 29 | 0·2 | |
| | Present | 8 | 8 | | |

TABLE I (*cont.*)

| Variable | Level | No. of patients | Median remission duration (months) | $P$ | Reference |
|---|---|---|---|---|---|
| Marrow reticulin (n = 83) | Normal | 28 | 41 | 0·2 | Hann *et al.*, 1978 |
| | Increased | 55 | 14 | | |
| % Cells in S phase (n = 44) | < 5 | 18 | 34 | 0·3 | Scarffe *et al.*, 1980 |
| | 6+ | 26 | 15 | | |
| Lymph-node size (n = 205) (cm) | < 1 | 39 | 36 | 0·3 | |
| | 1–2 | 119 | 34 | | |
| | 3+ | 47 | 15 | | |
| Weight percentile (n = 208) | 3 | 22 | 31 | 0·3 | |
| | 10 | 23 | 20 | | |
| | 25 | 62 | 18 | | |
| | 50 | 56 | 39 | | |
| | 75 | 24 | 25 | | |
| | 90 | 17 | 60+ | | |
| | 97 | 4 | 42 | | |
| Racial group | Caucasian | 194 | 33 | 0·3 | |
| | Asian | 9 | 18 | | |
| | Other | 6 | 10 | | |
| Platelets ($\times 10^9$/l) | < 25 | 106 | 25 | 0·4 | |
| | 25–50 | 48 | 31 | | |
| | 50–100 | 35 | 42 | | |
| | 100+ | 20 | 22 | | |
| % Marrow blasts | < 60 | 14 | 43 | 0·4 | |
| | 60–80 | 28 | 44 | | |
| | 80+ | 167 | 23 | | |
| % PAS$^+$ lymphoblasts with fine granules and blocks (n = 202) | < 10 | 134 | 22 | 0·5 | Hann *et al.*, 1979*a* |
| | 10–19 | 32 | 38 | | |
| | 20+ | 36 | 29 | | |
| Height percentile (n = 208) | 3 | 15 | 39 | 0·5 | |
| | 10 | 24 | 35 | | |
| | 25 | 46 | 31 | | |
| | 50 | 59 | 19 | | |
| | 75 | 33 | 32 | | |
| | 90 | 21 | 22 | | |
| | 97 | 10 | 23 | | |
| % Blasts vacuolated | < 10 | 108 | 28 | 0·5 | Hann *et al.*, 1979*a* |
| | 10–19 | 28 | 15 | | |
| | 20–49 | 35 | 33 | | |
| | 50–74 | 18 | 60+ | | |
| | 75+ | 16 | 36 | | |
| Bone involvement (n = 163) | None | 23 | 26 | 0·2 | Hann *et al.*, 1979*b* |
| | Minimal | 33 | 38 | | |
| | 1–2 | 26 | 17 | | |
| | 3–5 | 19 | 11 | | |
| | 6+ | 62 | 39 | | |
| Bone pain | Nil | 146 | 26 | 0·6 | Hann *et al.*, 1979*b* |
| | Mild | 14 | 17 | | |
| | Moderate | 20 | 28 | | |
| | Severe | 29 | 43 | | |
| Sex | Boys | 122 | 34 | 0·7 | |
| | Girls | 87 | 26 | | |
| Urea (mM) | < 7 | 165 | 30 | 0·9 | |
| | 7+ | 44 | 33 | | |
| Serious infection | Absent | 181 | 32 | 1·0 | |
| | Present | 28 | 18 | | |
| % Oil Red O cytochemical stain (n = 96) | < 10 | 76 | 26 | 1·0 | Hann *et al.*, 1979*a* |
| | 10+ | 20 | 37 | | |

* Statsitically significant, $P < 0{\cdot}05$.
† Not available at diagnosis.

FIG. 1.—Effect of WBC ( $\times 10^9$/l) at diagnosis on duration of first remission. $P$ for trend $< 0{\cdot}0001$.

FIG. 2.—Effect of FAB morphological classification (L1, L2 or L3) on duration of first remission. $P < 0{\cdot}0001$.

FIG. 3.—Effect of % $PAS^+$ lymphoblasts with coarse granules and blocks on duration of first remission. $P = 0{\cdot}0002$.

TABLE II.—*Results of the multivariate regression analysis*

$$\lambda(t) = \lambda_0(t) \exp(\beta_1 Z_1 + \beta_2 Z_2 + \beta_3 Z_3)$$

| Variable | Regression coefficient | $P$ |
|---|---|---|
| $Z_1$ = log WBC | $\beta_1 = 0{\cdot}875$ | $< 0{\cdot}00001$ |
| $Z_2$ = FAB morphological classification 0 for L1, 1 for L2 or L3 | $\beta_2 = 0{\cdot}709$ | $< 0{\cdot}001$ |
| $Z_3$ = log 1 + % $PAS^+$ lymphoblasts with coarse granules and blocks | $\beta_3 = -0{\cdot}389$ | $< 0{\cdot}002$ |

TABLE III.—*Prognosis in risk groups*

| Risk group | Risk score | Number of children (%) | Observed median duration of first remission (mths) | % still in first remission at 2 years |
|---|---|---|---|---|
| I | 1 | 69 (35) | 112+ | 81 |
| II | 2 | 56 (28) | 38 | 61 |
| III | 3 | 48 (24) | 10 | 32 |
| IV | 4 or 5 | 28 (14) | 8 | 11 |

Each patient's risk score (see Appendix) was calculated and the range of scores was from $-1$ to $3{\cdot}2$. For routine use the risk score is difficult to calculate, but an alternative approximate score can be easily found by using the following simple rule. (The equivalence of the exact and approximate scoring systems is shown in the Appendix.)

| | | |
|---|---|---|
| *WBC*: | $< 20 \times 10^9$/l | add 1 |
| | $20\text{–}50 \times 10^9$/l | add 2 |
| | $\geqslant 50 \times 10^9$/l | add 3 |
| *FAB type*: | L2 or L3 | add 1 |
| *PAS*+ | less than 5% | add 1 |

Four risk groups were formed, consisting of children with total scores of 1, 2, 3 and 4 or 5 respectively. The numbers of children in each risk group are shown in Table III, which also shows the observed median durations of first remission and the percentage in each group still in remission 2 years after remission induction. No more than one-third of patients fell into any one risk group and, by design, patients within each group have a similar prognosis. The value of forming risk groups is also demonstrated by the remission curves shown in Fig. 4.

FIG. 4.—Duration of first remission in each risk group.

To illustrate the use of the prognostic scoring system, consider one of the children in this study (MR), a boy aged 7 years who had a moderately high initial WBC of $15{\cdot}8 \times 10^9$/l at diagnosis. His disease was classed L2 according to the FAB morphological classification and 2% of his lymphoblasts contained PAS+ coarse granules or blocks. His risk score would therefore be 1 for WBC + 1 for FAB classification + 1 for % PAS positivity = 3. It is relevant to note that in fact he was in complete remission for only 5 months, and although a second remission was induced he died a year later. If the conventional criterion of WBC less than or more than $20 \times 10^9$/l had been used, this boy would have fallen into the "good risk" group. By contrast, SS is a girl aged 4 years who had an initial WBC of $44 \times 10^9$/l at diagnosis, L1 leukaemia, and 86% PAS+. In spite of her high initial WBC, which conventionally would put her into a "poor risk" group, her risk score is only 2, and her first remission lasted 6 years.

Potentially, the largest contribution to the total risk score comes from the initial WBC. This is not surprising since WBC is the most important single factor. However, its contribution may be outweighed by other factors. For example a child with L2 or L3 leukaemia and less than 5% of lymphoblasts with PAS+ coarse granules or blocks would already have a score of 2, and even a low initial WBC (normally a very favourable prognostic feature) would be enough to place him in Risk Group III, which has an estimated median time in first remission of only 10 months.

### DISCUSSION

There are many published reports of prognostic factors in acute leukaemia (see *Seminars in Oncology*, 3 (3), 1976 for a recent review). Most involve small numbers of patients, too few factors analysed and failure to take into account interrelationships between factors. No previous studies have been population-based. Some have analysed children and adults together (*e.g.* Bernard *et al.*, 1975) and even acute myeloid leukaemia with ALL, despite the known differences between these diseases (Gehan *et al.*, 1976). We investigated an unselected population-based group of children with ALL who received full conventional treatment. including CNS prophylaxis, at one institution under the care of one physician (PMJ). This study should reflect the heterogeneity of the disease and help to clear up much of the controversy over some prognostic factors.

Most published reports have shown that the prognostic factor of prime importance in ALL is a measurement of leukaemic mass, usually represented by the WBC and degree of organomegaly (Simone, 1975). The association of these factors with each other is also well known, but the fact that other mass factors have no separate prognostic significance when correlations with WBC are taken into account has not been fully appreciated. We have shown that although hepatomegaly and splenomegaly achieve prognostic significance when assessed individually, they have no independent significance in a multivariate analysis which includes WBC. Immunological blast surface markers also have a significant effect on prognosis when examined in isolation in our series, and in others (Greaves *et al.*, 1977; Chessells *et al.*, 1977), but this significance was not maintained in a multivariate analysis, because of the close association between T-cell disease and high WBC. Children with B-cell disease may well have very poor prognosis (Flandrin *et al.*, 1975) but there were too few patients in our series for separate analysis.

*FAB classification*

Various attempts have been made to classify ALL by morphological appearance (Mathé *et al.*, 1973; Flandrin & Bernard, 1975). The Franco–American–British classification (FAB, 1976) divides lymphoblasts into L1, L2 and L3 subtypes, the criteria being blast size as well as cytoplasmic and nuclear features. The L3 type corresponds to B-cell leukaemia, but there has been no consistent relationship between L1, L2 and other immunological subtypes (Tsukimoto *et al.*, 1976). We found the L2 subtype had larger blasts than L1 and a higher percentage of cells in S phase (Hann *et al.*, 1979*a*). These patients may thus have a larger tumour growth fraction, and this may be the reason for the worse prognosis in the L2 subtype.

*PAS score*

PAS score has previously been shown to be a significant factor in the prognosis of childhood ALL (Willoughby & Laurie, 1968; Lilleyman *et al.*, 1979; Hann *et al.*, 1979*a*). We found an association between low PAS score, L2 subtype and mediastinal mass, providing further evidence of a "sarcomatous" type of leukaemia.

In spite of a correlation with WBC as well, the multivariate analysis showed that the percentage of lymphoblasts with $PAS^+$ coarse granules and blocks was a significant determinant of remission duration which acted independently of other prognostic variables.

*Risk score*

We have constructed a simple risk score at diagnosis of childhood ALL based on the FAB classification, WBC, and % $PAS^+$ coarse granules or blocks. This risk score accurately predicts the subsequent prognosis of children who achieve a complete remission while the categories for WBC ($<20\times10^9$/l, $20–50\times10^9$/l and $\geqslant 50\times10^9$/l) conform to the usual clinical subdivisions of this variable.

For routine clinical use it is probably preferable to combine patients with risk scores 4 and 5 into a single Group IV. The largest group, Group I, contains only about one third of children. The observed median duration of first remission varied from only 8 months in Group IV to more than 9 years in Group I. This remarkable 13-fold difference in prognosis is probably a little exaggerated, because the scoring system which is optimal for this series is unlikely to be optimal in other series.

However, when used in other series the predictive power of the score should still be very high.

*Risk score in clinical trial design*

There are 3 applications of our risk score in clinical trial design. Firstly, many trials in childhood ALL are designed specifically for "good-" or "bad-" prognosis patients, assignment usually being to the latter group if the WBC is over $20 \times 10^9/l$ or if mediastinal mass is present. Our results have shown however that the effect of a favourable or unfavourable WBC can be outweighed by other factors, and therefore a subdivision of patients based on the risk score would be more likely to produce groups consisting of patients with roughly the same prognosis. The risk scores we have demonstrated should therefore be of value in identifying more accurately patients with good and bad prognosis for whom different trials may be designed; as when some poor-prognosis patients might be randomized to receive novel types of treatment, while some good-prognosis patients might receive less-toxic therapy.

Secondly, greater comparability of treatment groups has been achieved in many trials through randomization stratified by important prognostic variables. If stratification is used at all, practical reasons usually restrict it to one or at the most 2 variables, whereas our analysis has shown that in childhood ALL 3 prognostic variables are required to explain all the significant variation in remission times. Because randomization stratified by risk group is equivalent to stratification by the 3 prognostic variables which comprise the risk score, it provides a simple and effective means of ensuring that treatment groups are as similar as possible in all important respects.

Lastly, in deciding what variables to record at diagnosis of ALL, it is suggested that WBC, FAB classification, % $PAS^+$ and a few other key variables such as age, sex, serum immunoglobulin levels and surface markers should suffice. Most other variables may be dispensed with in the interests of simplicity.

*Risk score in clinical trial analysis*

Adjustment of a treatment comparison (or any other) to remove the simultaneous influence of other prognostic variables is one of the most useful features of the log-rank method (Peto *et al.*, 1977). In practice, adjustment for more than 3–4 variables is difficult and inefficient. However, adjustment by the single variable, risk group, is equivalent to simultaneous adjustment for the 3 prognostic variables of which the risk score is comprised, while being much simpler to carry out. Finally, the risk groups we have defined can be used to see whether a difference in outcome between two treatment groups in a clinical trial is the same in all sub-groups of patients. For example, the difference might have one value in the good-prognosis patients and another (perhaps even in the reverse direction) in the patients with bad prognosis. A simple and effective way in which this can be done is to examine the treatment difference within each risk group separately.

*Clinical use of the risk score*

The risk score is simple and quick to work out and gives an accurate prediction of outcome. It is hoped that it will be of great value to clinicians advising parents of a newly diagnosed child with ALL.

We wish to record our thanks to Mrs R. Hannon for typing the manuscript.

### APPENDIX

At any time, *t*, after remission induction a certain number of patients may relapse. The hazard function at time *t* is defined as the chance of relapsing on Day *t* among patients in remission on Day *t*. An undefined hazard function $\lambda_0(t)$ is taken to represent a standard hazard function, and the assumption of the Cox regression model is that the hazard function $\lambda(t)$ corresponding to a set ($z_1$, $z_2$, . . ., $z_p$) of values of the prognostic variables is then just a multiple of the standard hazard function. This multiple is $e^a$ where $a = Z_1\beta_1 + Z_2\beta_2 + \ldots + Z_p\beta_p$ and so the form of the Cox regression model is $\lambda(t) = e^a \lambda_0(t)$.

Each regression coefficient, $\beta$, in the equation reflects the net effect of the corresponding variable Z on the hazard function $\lambda_0(t)$ after the effects of all other variables have been accounted for. For each patient the value of $Z_1\beta_1+Z_2\beta_2+\ldots+Z_p\beta_p$ can be regarded as a "risk score", the higher the value the greater being the probability of relapse and the worse the prognosis.

Examination of the regression coefficients and variables in the risk-score equation suggested that a much simpler scoring system might have similar predictive power. It was found empirically that the simple risk score described in the text is for most children in the series the nearest integer to $1+1{\cdot}6\times$ exact risk score, while the limits used for allocating children to the different risk groups conform to well established clinical subdivisions. The logrank $\chi^2$ for trend obtained by comparing actual prognosis in the four risk groups was statistically highly significant ($\chi^2=61{\cdot}07$, d.f. = 1, $P \ll 0{\cdot}00001$) and was consistent with the overall increase in log-likelihood ($30{\cdot}87$, $\chi^2=61{\cdot}74$, d.f. = 3, $P \ll 0{\cdot}00001$) obtained in the multivariate regression analysis.

### REFERENCES

Bernard, J., Weil, M. & Jacquillat, C. (1975) Prognostic factors in human acute leukaemia. *Adv. Biosci.*, **14**, 97.

Byar, D. P., Green, S. B., Dor, P. & 6 others (1979) A prognostic index for thyroid carcinoma. A study of the EORTC thyroid cancer cooperative group. *Eur. J. Cancer*, **15**, 1033.

Chessells, J. M., Hardisty, R. M., Rapson, N. T. & Greaves, M. F. (1977) Acute lymphoblastic leukaemia in children: Classification and prognosis. *Lancet*, ii, 1307.

Cornbleet, M. A. & Chessells, J. M. (1978) Bone marrow relapse in acute lymphoblastic leukaemia in childhood. *Br. Med. J.*, ii, 104.

Cox, D. R. (1972) Regression models and life tables. *J. R. Stat. Soc. B.*, **34**, 187.

Draper, G., Birch, J., Bithall, J. & 6 others (1980) *Childhood Cancer in Britain* 1953–1975. London: HMSO (on behalf of OPCS). (In press.)

Flandrin, G. & Bernard, J. (1975) Cytological classification of acute leukaemias. A survey of 1400 cases. *Blood Cells*, **1**, 7.

Flandrin, G., Brouet, J. C. & Daniel, M. T. (1975) Acute leukaemia with Burkitt's tumour cells. *Blood*, **45**, 183.

Franco–American–British (FAB) Cooperative Group (1976) Proposals for the classification of acute leukaemias. *Br. J. Haematol.*, **33**, 451.

Gehan, E. A., Smith, T. L., Freireich, E. J. & 4 others (1976) Prognostic factors in acute leukaemia. *Semin. Oncol.*, **3**, 271.

Greaves, M. F., Janossy, G., Roberts, M. & 5 others (1977) Membrane phenotyping: Diagnosis, monitoring and classification of acute lymphoid leukaemia. In *Immunological Diagnosis of Leukaemia and Lymphoma.* Berlin: Springer-Verlag. p. 61.

Hann, I. M., Evans, D. I. K., Marsden, H. B., Morris-Jones, P. H. & Palmer, M. K. (1978) Bone marrow fibrosis in acute lymphoblastic leukaemia of childhood. *J. Clin. Pathol.*, **31**, 313.

Hann, I. M., Evans, D. I. K., Palmer, M. K., Morris-Jones, P. H. & Howarth, C. (1979*a*) The prognostic significance of morphological features in childhood acute lymphoblastic leukaemia. *Clin. Lab. Haematol.*, **1**, 215.

Hann, I. M., Gupta, S., Palmer, M. K. & Morris-Jones, P. H. (1979*b*) The prognostic significance of radiological and symptomatic bone involvement in childhood ALL. *Med. Paediatr. Oncol.*, **6**, 51.

Hann, I. M., Lees, P. D., Palmer, M. K., Gupta, S. & Morris-Jones, P. H. (1981) Renal size as a prognostic factor in childhood lymphoblastic leukaemia. *Cancer* (in press).

Hann, I. M., Morris-Jones, P. H., Evans, D. I. K., Addison, G. M., Palmer, M. K. & Scarffe, J. H. (1980) Low IgG or IgA: A further indicator of poor prognosis in childhood ALL. *Br. J. Cancer*, **41**, 317.

Hughes, W. T. (1971) Fatal infections in childhood leukaemia. *Am. J. Dis. Child.*, **122**, 283.

Kumar, S., Carr, T. F., Evans, D. I. K., Morris-Jones, P. & Hann, I. M. (1979) Prognostic significance of cell surface markers in childhood acute lymphoblastic leukaemia. *Clin. Lab. Haematol.*, **1**, 121.

Lendon, M., Hann, I. M., Palmer, M. K., Shalet, S. M. & Morris-Jones, P. H. (1978) Testicular histology after combination chemotherapy in childhood acute lymphoblastic leukaemia. *Lancet*, ii, 439.

Lilleyman, J. S., Mills, V., Sugden, P. J. & Britton, J. A. (1979) Periodic acid-Schiff reaction and prognosis in lymphoblastic leukaemia. *J. Clin. Pathol.*, **32**, 158.

Mathé, G., Pouillart, P., Weiner, R., Hayat, M., Steresio, M. & Lafleur, M. (1973) Classification and subclassification of acute leukaemia correlated with clinical expression, therapeutic sensitivity and prognosis. In *Recent Results in Cancer Research.* New York: Springer-Verlag. p. 6.

Miller, D. R., Leikin, S., Albo, V. & 4 others (1978) New prognostic factors in childhood leukaemia. *Paediatr. Res.*, **12**, 469.

Peto, R., Pike, M. C., Armitage, P. & 7 others (1977) Design and analysis of randomised trials requiring prolonged observation of each patient II: Analysis and examples. *Br. J. Cancer*, **35**, 1.

Scarffe, J. H., Hann, I. M., Evans, D. I. K. & 4 others (1980) Relationship between the pretreatment proliferative activity of bone marrow blast cells and prognosis of ALL of childhood. *Br. J. Cancer*, **41**, 764.

Simone, J. V. (1975) Prognostic factors in childhood acute lymphatic leukaemia. *Adv. Biosci.*, **14**, 27.

Tsukimoto, I., Wong, K. Y. & Lampkin, B. C. (1976) Surface markers and prognostic features in acute lymphoblastic leukaemia. *N. Engl. J. Med.*, **294**, 245.

Willoughby, M. L. N. & Laurie, H. C. (1968) The effect of cyclical maintenance therapy on first remission in acute leukaemia of childhood. *Arch. Dis. Child.*, **43**, 187.